# Serum Levels of miR-148a and miR-21-5p Are Increased in Type 1 Diabetic Patients and Correlated with Markers of Bone Strength and Metabolism

**DOI:** 10.3390/ncrna4040037

**Published:** 2018-11-27

**Authors:** Giuseppina E. Grieco, Dorica Cataldo, Elena Ceccarelli, Laura Nigi, Giovanna Catalano, Noemi Brusco, Francesca Mancarella, Giuliana Ventriglia, Cecilia Fondelli, Elisa Guarino, Isabella Crisci, Guido Sebastiani, Francesco Dotta

**Affiliations:** 1 Department of Medicine, Surgery and Neurosciences, University of Siena, 53100 Siena, Italy; giusy.grieco.90@gmail.com (G.E.G.); ele.ceccarelli77@gmail.com (E.C.); launigi@gmail.com (L.N.); giovanna_c90@libero.it (G.C.); noemibrusco91@gmail.com (N.B.); francescamancarella90@gmail.com (F.M.); giulianaventriglia@gmail.com (G.V.); francesco.dotta@alice.it (F.D.); 2 Fondazione Umberto Di Mario ONLUS c/o Toscana Life Science, 53100 Siena, Italy; 3 UOC Diabetologia, Azienda Ospedaliera Universitaria Senese, 53100 Siena, Italy; alexdori@libero.it (D.C.); c.fondelli@tin.it (C.F.); eguarino70@gmail.com (E.G.); crisci.isa@gmail.com (I.C.)

**Keywords:** circulating microRNAs, type 1 diabetes, bone metabolism, miR-148a

## Abstract

Type 1 diabetes (T1D) is characterized by bone loss and altered bone remodeling, resulting into reduction of bone mineral density (BMD) and increased risk of fractures. Identification of specific biomarkers and/or causative factors of diabetic bone fragility is of fundamental importance for an early detection of such alterations and to envisage appropriate therapeutic interventions. MicroRNAs (miRNAs) are small non-coding RNAs which negatively regulate genes expression. Of note, miRNAs can be secreted in biological fluids through their association with different cellular components and, in such context, they may represent both candidate biomarkers and/or mediators of bone metabolism alterations. Here, we aimed at identifying miRNAs differentially expressed in serum of T1D patients and potentially involved in bone loss in type 1 diabetes. We selected six miRNAs previously associated with T1D and bone metabolism: miR-21; miR-24; miR-27a; miR-148a; miR-214; and miR-375. Selected miRNAs were analyzed in sera of 15 T1D patients (age: 33.57 ± 8.17; BMI: 21.4 ± 1.65) and 14 non-diabetic subjects (age: 31.7 ± 8.2; BMI: 24.6 ± 4.34). Calcium, osteocalcin, parathormone (PTH), bone ALkaline Phoshatase (bALP), and Vitamin D (VitD) as well as main parameters of bone health were measured in each patient. We observed an increased expression of miR-148a (*p =* 0.012) and miR-21-5p (*p =* 0.034) in sera of T1D patients vs. non-diabetic subjects. The correlation analysis between miRNAs expression and the main parameters of bone metabolism, showed a correlation between miR-148a and Bone Mineral Density (BMD) total body (TB) values (*p =* 0.042) and PTH circulating levels (*p =* 0.033) and the association of miR-21-5p to Bone Mineral Content-Femur (BMC-FEM). Finally, miR-148a and miR-21-5p target genes prediction analysis revealed several factors involved in bone development and remodeling, such as MAFB, WNT1, TGFB2, STAT3, or PDCD4, and the co-modulation of common pathways involved in bone homeostasis thus potentially assigning a role to both miR-148a and miR-21-5p in bone metabolism alterations. In conclusion, these results lead us to hypothesize a potential role for miR-148a and miR-21-5p in bone remodeling, thus representing potential biomarkers of bone fragility in T1D.

## 1. Introduction

Risk of bone fractures is significantly higher in Type 1 diabetes (T1D) respect to the non-diabetic general population [1]. Indeed, prevalence of hip fractures in T1D patients have been reported to be up to six-fold higher than in the general population and 2.5-fold higher respect to type 2 diabetic (T2D) patients, thus suggesting the presence of specific and critical bone remodeling alterations in autoimmune diabetes [1,2].

One of the main parameters used to measure bone health and structure is represented by bone mineral density (BMD). Interestingly, while BMD was found significantly decreased in T1D patients [3,4], it is often normal or even increased in T2D patients [5,6], indicating the complex interplay among mechanisms characterizing T1D, T2D, and bone alterations.

The prominent increase of bone fractures and bone fragility in T1D is characterized by multiple determinants. For example, pro-inflammatory cytokines, which are major mediators of several diabetic complications, interfere with osteoblast differentiation and bone matrix collagen deposition [7], and contribute to increased bone microvascular dysfunctions [8], thus leading to an altered bone remodeling. In the same way, other common determinants, such as adipokines and bone marrow adiposity [9], hyperglycemia [10], and/or insulin deficiency [11,12], lead to a lower bone turnover, thus increasing the risk of fractures in diabetic patients.

An additional class of molecules potentially involved in the development of many diabetic complications is represented by microRNAs (miRNA). miRNAs are endogenous non-coding small RNAs, 19–24 nucleotides long, which negatively regulate gene expression through their specific binding to 3’UTR region of messenger RNA [13]. miRNAs have been reported to regulate many cellular processes through the modulation of entire signaling pathways. Consequently, alterations of several miRNAs, reported in different diseases, can surely contribute to define a disease phenotype. As a matter of fact, specific miRNA alterations have been associated to T1D both in pancreatic islets [14] and in immune cells [15,16], thus characterizing diabetes outcome [15,17]. Additionally, miRNAs have been found altered in several tissues affected by typical secondary diabetic dysfunctions (e.g., vascular endothelium-miR-126) [18]. Moreover, miRNAs participate in several mechanisms involved in bone development and function, modeling and remodeling, and characterize the phenotype of bone cells, including osteocytes, osteoblasts, and osteoclasts [19]. Of note, miRNAs have been demonstrated to be involved also in bone diseases [20].

Recent studies demonstrated that miRNAs are not confined within cells of origin but can be also secreted, thus being found in different biological fluids such as serum or plasma [21]. In serum/plasma they can be found associated with different cellular components such as protein-bound miRNAs (Ago2 or HDL) or within extracellular vesicles (EVs) (e.g., exosomes) [21]. Although the significance of specific miRNA secretion in biological fluids has not been clearly defined yet, several studies demonstrated that: (*i*) blood circulating miRNA expression profiles are altered in a variety of diseases [22]; (*ii*) alterations of multiple circulating miRNAs can define a tissue-specific dysfunction thus being potentially used as disease biomarkers [23]; and (*iii*) circulating miRNAs are not only byproducts of cell loss but have been reported to be active molecules which mediate communication among different tissues and cells [24]. For these reasons, miRNAs may represent both optimal biomarkers of specific tissue dysfunction and potential molecules orchestrating distant molecular mechanisms [25]. Therefore, the identification of an alteration of a specific set of circulating miRNAs may pave the way for the discovery of new biomarkers and for the identification of new therapeutic targets [26].

Here, we hypothesize that a specific set of circulating miRNAs, found altered in serum/plasma of T1D patients, may represent also optimal biomarkers of bone fragility and/or mediators of bone tissue dysfunctions, given their previously reported role both in autoimmune diabetes and in bone homeostasis, in the regulation of osteoclastogenesis, osteoblastogenesis, and their modulation.

## 2. Results

### 2.1. A Specific Set of miRNAs Is Altered in T1D and Involved in Bone Metabolism

An extended literature search was initially performed in order to identify a specific set of candidate circulating miRNAs whose expression has been previously associated with T1D and which have also been proposed as modulators of bone homeostasis, being involved in bone development, modeling and/or remodeling. Then, we looked for their previously reported role in bone cells/metabolism. We selected six miRNAs, all significantly dysregulated in serum or plasma of T1D patients, namely: miR-148a [27,28,29,30], miR-21-5p [28,29], miR-214 [31], miR-375 [28,29,32], miR-27a [27], and miR-24 [27,28,29,31,32]. All have a clear and demonstrated role in osteoblastogenesis or osteoclastogenesis, thus being involved in function of osteoblasts or osteoclasts, or found altered in serum/plasma of patients with bone diseases (e.g., osteoporosis) (Table 1) [33,34,35,36,37,38,39,40]. Above mentioned miRNAs were taken into consideration for further expression analysis in sera collected from a selected cohort of T1D patients and control subjects who were previously characterized in terms of parameters of bone homeostasis and bone strength.

### 2.2. CirculatingLevels of miR-148a and miR-21-5p Are Increased in Sera of T1D Patients vs. Control Subjects

In order to analyze the expression of the selected miRNAs reported in Table 1, we took into consideration a specific cohort of T1D patients and non-diabetic volunteers, already characterized for a specific panel of bone metabolic markers and bone physical parameters. T1D patients and healthy subjects were further selected based on age and gender, in order to perfectly match the two groups (Table 2 and Table S1). Of note, as previously reported by our group [41] and in line with several studies published by others [42,43], T1D patients showed a significant reduction of Bone Mineral Density-Femur (BMD-FEM), Bone Mineral Content-Femur (BMC FEM), T score FEM and Z score FEM as well as Bone Mineral Density-Neck (BMD-N), T score N and Z score N vs. non-diabetic subjects, confirming the alterations of bone metabolism in T1D. Additionally, we observed a significant reduction of 25-hydroxy vitamin-D (Vit D) and a concomitant increase in parathormone (PTH) in T1D patients vs. non-diabetic individuals (Table 2). Collectively, these results highlight a reduction of bone strength in T1D patients.

**Figure 1:**
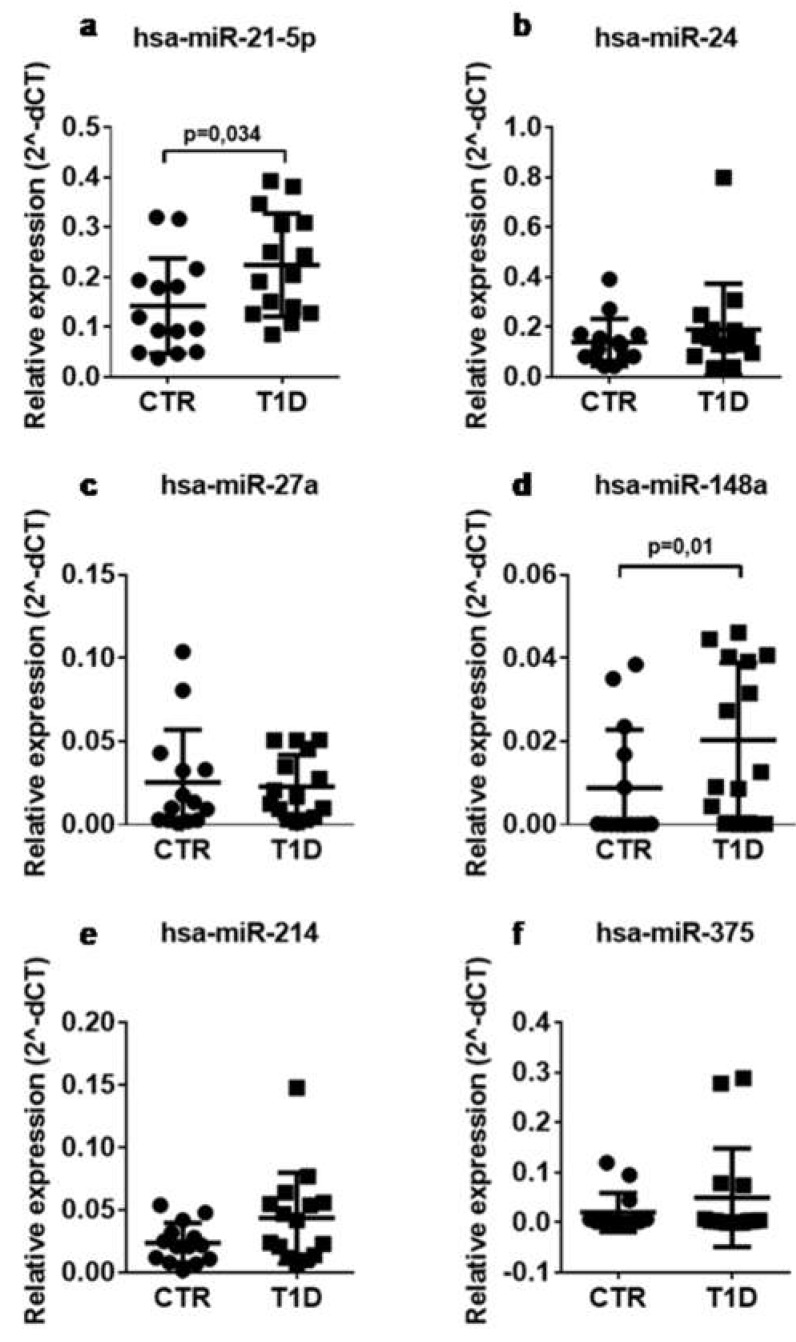
The expression of hsa-miR-148a and miR-21-5p is increased in the serum of patients with T1D. Single assay RT-qPCR validation of miR-21-5p (**a**), miR-24 (**b**), miR-27a (**c**), miR-148a (**d**), miR-214 (**e**), and miR-375 (**f**) in *n* = 14 non-diabetic and *n* = 15 T1D patients. Data are reported as mean ± SD of normalized 2^−ΔCT^ values. Statistics using Mann–Whitney U test, *p* < 0.05.

Serum samples from the same cohort of T1D patients (*n* = 15) and non-diabetic controls (*n* = 14) were subjected to expression analysis of the 6 selected miRNAs, namely miR-148a, miR-21-5p, miR-214, miR-375, miR-27a, and miR-24, using RT-Stem Loop Real Time PCR. Among selected miRNAs, miR-148a and miR-21-5p expression levels resulted significantly increased in sera of T1D patients vs. non-diabetic controls, while no significant difference was observed for other miRNAs (Figure 1).

### 2.3. Circulating miR-148a and miR-21-5p Levels Are Associated with BMD-Total Body (TB), PTH, and BMC-FEM

**Figure 2:**
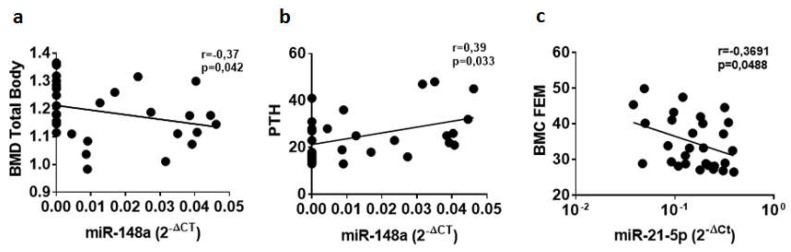
The expression of hsa-miR-148a and miR-21-5p levels are correlated with bone metabolism parameters in T1D patients and non-diabetic control subjects. Correlation analysis between miR-148a serum expression levels, reported as normalized 2^−ΔCT^ values, and BMD total body (TB) reported as g/cm^2^ (**a**) and circulating levels of parathormone (PTH) reported as pg/mL (**b**). Correlation analysis between miR-21-5p serum expression levels and BMC-FEM reported as g/cm^2^ (**c**). Spearman *R* test was performed to evaluate *r*-values and *p*-values (*p* < 0.05).

In an attempt to get insight into the potential role of selected circulating microRNAs in bone metabolism, we correlated their expression levels to the main parameters of bone homeostasis and metabolism measured in T1D patients and non-diabetic subjects. Initially, we took into consideration miR-148a and miR-21-5p due to their peculiar differential expression in sera of T1D patients. No significant correlations were identified for miR-148a and miR-21-5p respect to the main clinical T1D patient characteristics (age, BMI, disease duration and autoantibodies positivity) (Figure S1). Conversely, miR-148a correlation analyses with bone metabolic parameters (Figure S2 and Table S2), showed a significant negative correlation between serum miR-148a and BMD-TB (Figure 2a), thus suggesting a potential association of miR-148a with the regulation of bone strength (Figure 2a). Concomitantly, miR-148a was also positively correlated with circulating PTH (Figure 2b), reinforcing the potential link between miR-148a and bone metabolism. Additionally, a significant correlation between miR-21-5p and BMC-Fem was observed as well (Figure 2c), thus highlighting the potential role of miR-21-5p in the modulation of bone strength, in line with miR-148a.

Finally, the correlation analysis between selected miRNAs and parameters of bone metabolism revealed a positive significant association of miR-375 expression levels with PTH (Supplementary Figure S2).

### 2.4. Bioinformatic Analysis Revealed that miR-148a and miR-21-5p Target Several Genes Involved in Bone Metabolism and Bone Remodeling

Given the observed correlation between miR-148a and miR-21-5p with bone strength parameters and PTH levels, we aimed at evaluating a potential mechanism in which this miRNAs could be involved in the modulation of bone modeling and/or function. To this purpose we looked for predicted and experimentally validated miR-148a and miR-21-5p target genes which could directly or indirectly modulate bone metabolism. Using two manually curated databases (Tarbase and miRTarbase) exclusively containing experimentally validated target genes, and revising previous published studies, we detected several miR-148a and miR-21-5p target genes reported to be involved in bone functions and remodeling (Table 3). Among these, we identified MAFB [44] and WNT1 [45,46] which have been reported to be pivotal modulators of bone remodeling through the regulation of osteoclasts [44] and osteoblasts [47] differentiation, respectively, thus suggesting a potential role for miR-148a in the regulation of bone resorption and deposition in physiologic conditions and in T1D. Additionally, miR-21-5p has been reported to target several genes involved in bone remodeling; among them, we identified STAT3 ascribed as a pivotal modulator of bone metabolism and remodeling [48,49] (Table 3).

**Figure 3:**
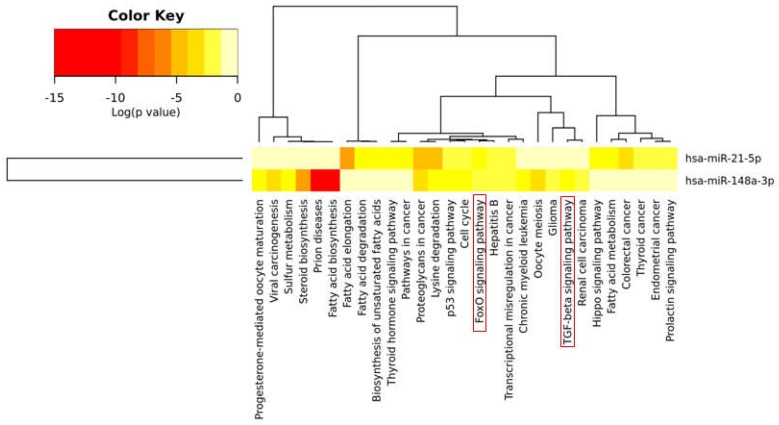
miR-148a and miR-21-5p regulate common pathways involved in bone metabolism and remodeling. Hierarchical Clustering Heatmap bioinformatic analysis of Kyoto Encyclopedia of Genes and Genomes (KEGG) pathways of hsa-miR-148a-3p and hsa-miR-21-5p target genes, showed FoxO and TGF-β signaling pathway as main pathways involved in bone metabolism and remodeling. Of note, FoxO signaling pathway is commonly identified both in miR-148a-3p and miR-21-5p analysis. Color key indicates Log *p* values from the less significant pathway (**light yellow**) to the most significant pathway (**dark red**).

Furthermore, the Kyoto Encyclopedia of Genes and Genomes (KEGG) pathway and Gene Ontology (GO) analysis, performed through the use of Diana tool miRPath v3.0 software and aimed at identifying miR-148a and miR-21-5p common regulated pathways, identified 1372 validated target genes for hsa-miR-21-5p and 1400 validated target genes for hsa-miR-148a. Most importantly, 24 validated target genes of miR-21-5p and 28 validated target genes of miR-148a-3p are commonly involved in FoxO signaling pathway (*p =* 0.00087). Moreover, 15 validated target genes of miR-148a-3p are also involved in TGF-β signaling pathway (*p =* 0.0051) (Figure 3 and Supplementary Table S3). Of note, both FoxO [50,51,52,53,54] and TGF-β [55,56,57,58] signaling pathways have been largely reported to be involved in bone metabolism, resorption and remodeling thus highlighting the putative role of miR-148a and miR-21-5p in the modulation of bone metabolism through the regulation of common pathways.

## 3. Discussion

To our knowledge, this is the first study which associates circulating miRNAs expression to bone metabolism alterations in type 1 diabetes (T1D). Multiple studies suggested that circulating miRNAs could be novel potential disease biomarkers [22], also critically involved in the regulation of molecular mechanisms at distant sites from their cells of origin [25]. Such properties make miRNAs ideal predictive, diagnostic and follow-up biomarkers, as well as putative therapeutic targets.

In T1D, several studies identified multiple alterations of circulating miRNAs, even though the results were in some cases discordant [17,26]. Of note, circulating miRNAs have been found associated with several life-threatening T1D complications, thus being suggested as potential therapeutic targets or as precious biomarkers for an early detection respect to the clinical onset of such complications [18,59,60].

We and others have previously demonstrated that T1D patients show an increased risk of fractures [61] due to peculiar alterations of bone metabolism/strength and remodeling [41]. Indeed, such patients were characterized by lower BMD and BMC, higher PTH levels and by an additional plethora of alterations of bone metabolism which highlighted a lower bone turnover, suggesting, in turn, an alteration of bone modeling and remodeling in autoimmune diabetes. However, although BMD represents a good indicator of risk of fractures, it could underestimate it, thus making risk assessment a challenging analysis. To this regard, the introduction of novel biomarkers, such as miRNAs, may help in risk assessment and, potentially, in an accurate prediction and evaluation of bone fragility. Moreover, among several potential causes of bone fragility that have been suggested, at present a role for circulating miRNAs in T1D as mediators of bone metabolic alterations has not yet been proposed.

As a matter of fact, in T1D as well as in bone diseases, miRNAs have been shown to be differentially expressed in tissues (e.g., pancreatic islet cells, immune cells, and bone cells) as well as in serum/plasma [20,29], thus underlining the importance of evaluating circulating miRNAs both as biomarkers and/or as causative/contributing factors to a specific disease.

In the present study, a set of miRNAs selected based on their reported involvement both in T1D pathogenesis and in bone metabolism/bone remodeling (miR-148a, miR-21-5p, miR-214, miR-375, miR-27a, and miR-24) were evaluated in serum samples of 15 T1D patients and of 14 age-matched control subjects; results showed and confirmed the hyperexpression of miR-148a and miR-21-5p in sera of T1D patients vs. non-diabetic individuals. miR-148a and miR-21-5p were previously observed as differentially expressed in T1D serum/plasma; in the present study such differential expression is confirmed and additionally associated to bone fragility and bone metabolism alterations in T1D, being inversely correlated with BMD-TB (miR-148a) and BMC-FEM (miR-21-5p) and positively associated with PTH levels (miR-148a).

Particularly, hyperexpression of miR-148a and miR-21-5p in serum/plasma from T1D patients were previously observed in several different studies which evaluated circulating miRNAs expression profiles in T1D patients cohort of similar composition (number of T1D patients and non-diabetic controls and clinical characteristics) with respect to the present study [27,28,29,30,32]. Of note, in two independent studies, miR-148a was also reported to be increased in plasma of osteoporotic (OP) postmenopausal women [33] and in sera from OP patients with reported hip fractures [34], thus suggesting a link between miR-148a, T1D and bone remodeling. Similarly, miR-21-5p was previously associated to bone remodeling [62] and its circulating expression signature was suggested as a potential biomarker of bone diseases [63]. The analysis of clinical characteristics of T1D patients led us to exclude a role for some major confounders (e.g., diabetic complications, other inflammatory diseases, etc.—see Table S1) in dictating miR-148a and miR-21-5p expression levels, since no differential expression were detected as associated with these clinical characteristics (Supplementary Table S1).

Although such results are in line to what was previously reported and demonstrated for miR-148a and miR-21-5p expression in T1D and in bone diseases, our data should be further confirmed in additional patients and controls thus aiming at reinforcing such results. Similarly, the correlations between miR-148a and miR-21-5p with parameters of bone metabolism should be performed and confirmed by taking into consideration an extended cohort of patients. In fact, although the associations of such miRNAs to bone alterations interestingly suggest their potential involvement in bone homeostasis, our cohort did not reach the statistical power to unequivocally correlate them exclusively in T1D patients or in non-diabetic control cohort, thus representing a limit of our study. On the other hand, our results indicate that miR-148a and miR-21-5p are significantly associated to parameters of bone metabolism in T1D patients and non-diabetic controls as a potential global effect of a physiological regulatory mechanism which could be exacerbated and altered in T1D patients due to hyperexpression of these circulating miRNAs. Of note, although the correlations between miR-148a/miR-21-5p and parameters of bone metabolism and strength did not reach statistical significance by analyzing only T1D group, such correlations showed an interesting tendency (data not shown) which should be further examined in future studies.

An additional potential issue regarding circulating miRNAs is the origin of their secretion. Indeed, although at present we cannot decipher the cells of origin of circulating miR-148a or miR-21-5p, a previously published study revealed that an increased expression of miR-148a occurred in CD14^+^ osteoclasts precursor cells derived from lupus patients [44]. Such observation suggests that circulating miR-148a alteration might represent a common phenomenon occurring in autoimmune diseases which potentially affects bone strength. As a matter of fact, in the same study, it was demonstrated that miR-148a negatively regulates MAFB, which, in turn, negatively modulates Receptor Activator of Nuclear factor Kappa-Β Ligand (RANKL) induced osteoclastogenesis, thus resulting in an increased rate of osteoclasts differentiation and, consequently, elevated bone resorption. Collectively, these data suggest that the increased circulating levels of miR-148a may derive from CD14^+^ osteoclast precursor cells, thus potentially explaining the inverse correlation between miR-148a and BMD, being the latter associated with an increased rate of bone resorption. Additionally, it has been observed that miR-148a expression is physiologically reduced during the differentiation of bone marrow derived mesenchymal stem cells (MSCs) towards osteogenic lineage [64] while its upregulation promoted adipogenic differentiation (by repressing WNT1) [46]; moreover, an increased expression of miR-148a was observed in exosomes derived from MSC cultures during osteogenic differentiation [65], showing miR-148a secretion and modulation during bone modeling. Altogether, these data suggest that in-situ miR-148a hyperexpression may be detrimental to bone modeling and remodeling and that miR-148a may be secreted through exosomes, thus being potentially detectable in biological fluids.

As for miR-21-5p, it has been previously extensively demonstrated its role in the modulation of osteoclastogenesis [66]. Of note, a recent report demonstrated that circulating miR-21-5p secreted through exosomes may control osteoclastogenesis [67], thus underlining the potential role of this circulating miRNA in the modulation of bone remodeling or as promising biomarker [68]. 

Collectively, these evidences suggest that miR-148a and miR-21-5p regulate common pathways involved in bone metabolism and remodeling.

In addition, we demonstrate that the expression levels of miR-148a are positively correlated with PTH. It has been shown that increased levels of miR-148a in rat parathyroid cultures induce an abnormal PTH secretion; importantly, a specific reduction of such miRNA, by using miR-148a antagomiRs, affected PTH secretion in vivo, thus suggesting an association between miR-148a levels and PTH serum concentration [69]. We can hypothesize that the increased circulating expression of miR-148a may have a broad range of effects, thus potentially modifying the secretion of PTH by targeting parathyroid cells as well. Although miR-148a has been reported to be an in vivo modulator of PTH secretion, we cannot exclude that the observed correlation between this miRNA and PTH may also derive from an indirect effect of PTH itself on bone metabolism, which may in turn alter miR-148a expression and secretion. Of note, we also identified miR-375 as positively correlated with PTH circulating levels, thus suggesting a role for miR-375 in the regulation of PTH in T1D patients and non-diabetic controls, even though its expression levels did not differ between the two groups analyzed.

In conclusion, in this study we confirmed the hyperexpression of circulating miR-148a and miR-21-5p in sera from T1D patients; additionally we reported the association of miR-148a hyperexpression to several markers of bone fragility. Further, we showed and discussed that miR-148a and miR-21-5p targets pivotal genes involved in bone remodeling pathways, thus raising the hypothesis of miR-148a/miR-21-5p as contributing factors to bone fragility or as a potential biomarkers of bone metabolic alterations in T1D.

## 4. Materials and Methods

### 4.1. Ethical Statement

All procedures followed were in accordance with the ethical standards of the responsible committee on human experimentation (institutional and national) and with the Helsinki Declaration of 1975, as revised in 2008. The research protocol was submitted for consideration, comment, guidance and approval to the concerned research ethics committee, independently on the researcher, the sponsor and any other undue influence and is duly qualified. Every law and regulation of the country in which the research has been performed has been taken into consideration. The study was approved by the local Institutional Review Board (Comitato Etico Regionale per la Sperimentazione Clinica della Regione Toscana—Prot: 11807_2017—16/10/2017), and written informed consent was obtained from all participants as previously reported [41].

### 4.2. Study Population

A total of *n* = 15 patients with type 1 diabetes (T1D) (age range 24–49 years, mean disease duration 15.71 ± 11.33 years) referred to the Diabetes Unit of our department were included in the study. All patients had normal serum creatinine levels and no major comorbidities impairing normal daily activity. All patients were positive for at least one autoantibody at the onset of T1D. All patients were on treatment with insulin. Main clinical characteristics are reported in Table 2 and extended Table S1 which includes the clinical details of T1D patients regarding diabetic complications (2/15 T1D patients reported retinopathy; neither micro- nor macro-albuminuria was detected), current medications, other inflammatory diseases, and smoking habits alongside with miRNAs expression levels for each patient analyzed.

Age- and sex-matched controls (*n* = 14) were recruited from healthy volunteers. All control subjects had normal fasting glucose levels and HbA1c, 5/14 reported smoking habits, no chronic inflammatory diseases or current medications were reported.

Subjects with Paget’s disease of bone, primary hyperparathyroidism, congestive heart failure, recent myocardial infarction, multiple myeloma, or other neoplasia were excluded from the study. Moreover, subjects were also excluded if they received treatment with anti-resorptive or anabolic compounds for osteoporosis and current corticosteroid therapy, or any other treatment known to affect bone metabolism.

General and clinical characteristics of patients and controls are reported in Table 2.

### 4.3. Samples Collections and Clinical Analysis

Blood samples were collected in the morning after an overnight fast, in serum gel tube (BD Vacutainer) and stored at room temperature for maximum 2h. Blood samples were then centrifuged 1800×*g* for 15min at 4 °C and then stored at −80 °C.

Serum calcium concentrations (Ca^2+^) (corrected for albumin concentration), were measured using standard automated laboratory techniques. Circulating levels of intact osteocalcin [DiaSorin Diagnostics (Saluggia, Italy); interassay CV 7.1%, normal range 1.8–6.6 ng/mL] and of bone-specific alkaline phosphatase (bALP) (Beckman Coulter, Fullerton, CA, USA; with an interassay CV of_7.9%, normal range 9–21_g/L) were measured in serum samples as markers of bone turnover. Circulating PTH (DiaSorin, Stillwater, MN, USA; interassay CV _7.3%; normal range 10–60 pg/mL) and 25OH Vitamin D (DiaSorin Diagnostics; sensitivity, 1.5 ng/mL; interassay CV _11%; normal range for vitamin D sufficiency, _30 ng/mL) were evaluated by RIA. 

At the time of blood sampling, areal BMD of the lumbar spine and of the proximal femur was determined by a dual-energy X-ray absorptiometry device (Lunar Prodigy; GE Healthcare, Waukesha, WI, USA).

### 4.4. RNA Extraction

Serum samples were thawed on ice and then further centrifuged 3000× *g* for 5 min at 4 °C in order to completely remove contaminant cells and cell debris. A total of 50 μL of serum from each patient was diluted in 350 μL of nuclease-free water to avoid protein aggregates. Then, RNA was extracted from diluted serum by using miRNeasy miRNA extraction kit (Qiagen, Hilden, Germany), by adding 1.2 mL of Trizol LS (Lifetechnologies, Carlsbad, CA, USA) as lysis buffer and finally eluted in 30 μL of nuclease-free water.

### 4.5. Single Assay qRT Real-Time PCR

The expression of miRNAs miR-21-5p, miR-24-3p, miR-214, miR-375, miR-27a and miR-148a was analyzed in all 29 serum samples through single assay qRT Real-Time PCR using TaqMan miRNA assay primers (Life technologies, CA, USA). RNA was reverse-transcribed employing Custom RT primers pool and preamplified using Custom Preamp primers pool. Briefly, 5 µL each RT or TM primer was diluted in a total volume of 500 µL Tris-EDTA 1X and used for RT or preamplification reaction. Then, 3 μL of extracted RNA were added to 6 μL of custom primers pool, 0.30 μL 100 mM dNTPs, 3 μL of 50 U/μL Multiscribe RT, 1.50 μL 10× RT Buffer, 0.19 μL 20 U/μL RNase Inhibitor and 1.01 μL H_2_O. The reaction product was incubated at 16 °C for 30 min, 42 °C for 30 min and then at 85 °C for 5 min. Afterwards, the synthesized cDNA was preamplified using Custom Preamp primer pool: 2.5 μL of cDNA from each sample were added to 12.5 μL 2× TaqMan Preamp Master Mix, 3.75 μL 10× Custom Preamp primers and 6.75 μL H_2_O. The reaction was incubated at 95 °C for 10 min, at 55 °C for 2 min and at 72 °C for 2 min, then for 12 cycles at 95 °C for 15 s and 60°C for 4 min and, finally, at 99 °C for 10 min. In each well, 5 μL of preamplified cDNA (diluted 1:8 in Tris-Edta 0.1X) were added to 15 μL reaction mix composed of 10 μL TaqMan Universal Master Mix, 1 μL of TaqMan miRNA expression assay and 4 μL of nuclease-free H_2_O. The reaction was incubated at 95 °C for 10 min, followed by 40 cycles at 95 °C for 15 s and at 60°C for 1min.

Data analysis was performed using 2^−Δ*C*t^ method; samples with resulting raw cycle-threshold (*C*_t_) >35.0 were considered as not detected/not expressed. Normalization analysis was performed by using miRNAs hsa-miR-451 to exclude hemolyzed samples alongside with 414 nm spectrophotometer absorbance [70,71,72], hsa-miR-191 as endogenous control previously reported by other studies [73,74,75] and exogenous ath-miR-159a as technical control of RNA extraction reproducibility [76,77].

### 4.6. Bioinformatic Analysis

Diana tool mirPath v.3 online software (http://snf-515788.vm.okeanos.grnet.gr/) was used to identify validated target genes of miR-148a and miR-21-5p. Furthermore, through the use of the same software, KEGG pathway and Gene Ontology (GO) analysis has been performed to address a putative role for these microRNAs in molecular pathways with a specific involvement in bone metabolism and remodeling.

### 4.7. Statistics

Data have been reported as means or median ± SD; sample values distribution analysis was performed for all parameters by using D’Agostino & Pearson normality test. Non-parametric Mann-Whitney U Test or Student’s *T*-test were adopted for *p*-value evaluation according to values distribution; a *p* value ≤0.05 was considered as statistically significant.

MicroRNAs expression reported as normalized 2^−ΔcT^ values were compared between T1D and the control group using Mann-Whitney U test (*p* value *≤* 0.05). Logistic regression and Spearman correlation analysis were performed to evaluate the association between miR-148a expression and measured bone parameters.

## Figures and Tables

**Table 1:** Selected miRNAs analyzed in the study.

Selected miRNAs	Evidences of Circulating miRNAs Differentially Expressed in T1D Patients	Evidences of miRNAs Involved in Bone Remodeling
hsa-miR-148a	Nielsen et al. 2012 [27] Seyan et al.2016 [28] Assman et al. 2017 [29] Akerman 2018 [30]	Bedene et al. 2016 [33] Seeliger et al. 2015 [34]
hsa-miR-21-5p	Seyan et al.2016 [28] Assman et al. 2017 [29]	Li et al. 2014 [35] Seeliger et al. 2015 [34] Yang et al. 2013 [36]
hsa-miR-214	Erener et al. 2017 [31]	Zhao et al. 2015 [37]
hsa-miR-375	Seyan et al.2016 [28]	Du et al. 2015 [38]
	Samandari et al.2016 [32]	
	Assman et al. 2017 [29]	
hsa-miR-27a	Nielsen et al.2012 [27]	You et al. 2016 [39]
		Zeng et al. 2017 [40]
hsa-miR-24	Seyan et al.2016 [28]	Seeliger et al. 2015 [34]
	Samandari et al.2016 [32]	
	Assman et al. 2017 [29]	
	Nielsen et al. 2012 [27]	
	Erener et al. 2017 [31]	

**Table 2:** Main clinical characteristics of non-diabetic subjects and type 1 diabetic patients

	Non-Diabetic Subjects	Type 1 Diabetic Patients
Subjects (*n*)	14	15
Male/Female (*n*/*n*)	10/4	10/5
Age (years)	28 ± 7.93	32 ± 7.9
Disease duration (years)	/	15.71 ± 11.33
HbA1c (%)	N/A	7.36 ± 0.80593
Chronic complications (*n*/*n*)	N/A	2/15
Smoking habits (*n*/*n*)	N/A	3/15
Other therapies (*n*/*n*)	N/A	6/15
Chronic inflammatory diseases (*n*/*n*)	N/A	4/15
BMI ^#^	24 ± 4.34	21 ± 1.73 ^#^
Ca (mg/dL)	9.37 ± 0.50	9.54 ± 0.46
Osteocalcin (ng/mL)	4.24 ± 1.82	4.50 ± 2.21
bALP (μg/L)	10.5 ± 7.33	10.2 ± 4.72
25OHD (ng/mL) ^#^	27.9 ± 10.33	14.3 ± 9.83 ^#^
PTH (pg/mL) ^#^	18 ± 9.36	26 ± 10.23 ^#^
BMD TB (g/cm^2^)	1.23 ± 0.11	1.15 ± 0.09
T score TB	0.7 ± 1.18	−0.2 ± 1.15
Z score TB	0.43 ± 1.10	0.08 ± 1.12
BMC TB (g/cm^2^)	2.82 ± 0.46	2.61 ± 0.38
BMD FEM (g/cm^2^) ^#^	1.156 ± 0.17	0.967 ± 0.13 ^#^
T score FEM ^#^	0.8 ± 1.18	−0.8 ± 1.10 ^#^
Z score FEM ^#^	0.6 ± 1.04	−0.8 ± 1.11 ^#^
BMC FEM (g/cm^2^) *	37.86 ± 8.13	32.04 ± 4.82*
BMD N (g/cm^2^) *	1.09 ± 0.18	0.94 ± 0.12*
T score N *	0.3 ± 1.27	−0.80 ± 1.08*
Z score N ^#^	0.2 ± 1.13	−0.8 ± 1.10 ^#^
BMC N (g/cm^2^)	5.34 ± 1.35	4.40 ± 1.04
BMD L (g/cm^2^)	1.20 ± 0.15	1.14 ± 0.17
T score L	−0.04 ± 1.24	−0.57 ± 1.48
Z score L	−0.14 ± 1.26	−0.42 ± 1.59

Note: BMI= Body Mass Index; Ca = Calcium; bALP = Bone Alkaline Phosphatase; 25 OHD = 25 OH Vitamin D; PTH = Parathormone; BMD = Bone Mineral Density; BMC= Bone Mineral Content; TB = Total Body; FEM= Femur; N = Neck; L = Lumbar. Samples distribution analysis was performed for all parameters by using D’Agostino & Pearson normality test. BMI, bALP, 25 OHD, PTH, T score TB, BMD FEM, T score FEM, Z score FEM and Z score N are indicated as median ± SD (^#^ Mann-Whitney U test = *p* ≤ 0.05); age, Ca, osteocalcin, BMD TB, Z score TB, BMC TB, BMC FEM, BMD N, T score N, BMC N, BMD L, T score L, Z score L are indicated as mean ± SD. (* Unpaired *t* test = *p* ≤ 0.05).

**Table 3:** Validated target genes of hsa-miR-148a and hsa-miR-21-5p.

Target Gene	Gene Name	Function	Targeting miRNA
***MAFB***	V-mafavianmusculoaponeurotic fibrosarcoma oncogenehomolog B	Negatively regulates RANKL-induced osteoclast differentiation	miR-148a
***WNT1***	Wingless-type MMTV integration site family, member 1	*Canonical pathway*Wnt/β catenin: stimulates osteoblast differentiation and proliferation	miR-148a
***BCL2L11***	BCL2-like 11 (apoptosis facilitator)	Induces apoptosis	miR-148a
***PTEN***	Phosphatase and tensin homolog	Interacts with *Canonical pathway*Wnt/β catenin	miR-148a
***TGFB2***	Transforming growth factor, β 2	Stimulates osteoblast differentiation and proliferation and bone formation	miR-148a
***IGF1***	Insulin-like growth factor 1 (somatomedin C)	Stimulates bone mineralization and positively regulates osteoblast differentiation	miR-148a
***GADD45A***	Growth arrest and DNA-damage-inducible, α	Positively regulates apoptosis	miR-148a
***STAT3***	Signal transducer and activator of transcription 3	Specifically inactivates osteoblast/osteocytes	miR-21-5p
***TGFBR1***	Transforming Growth Factor β Receptor 1	Regulates bone resorption	miR-21-5p
***IGF1R***	Insulin-like growth factor 1 receptor	Involved in bone remodeling	miR-21-5p
***BCL6***	B-Cell lymphoma 6	Regulates osteoclast differentiation	miR-21-5p
***TGFB2***	Transforming growth factor-β 2	Involved in osteoblast differentiation and bone remodeling	miR-21-5p
***PDCD4***	Programmed Cell Death 4	Involved in osteoclastogenesis through cFos regulation	miR-21-5p
***SOD2***	Superoxide dismutase 2	Controls osteoclastogenesis and maintains osteoblast differentiation	miR-21-5p
***FOXO3***	Forkhead box O-3	Its overexpression increases bone resorption	miR-21-5p
***USP7***	Ubiquitin-specific protease 7	Involved in osteogenic differentiation	miR-21-5p
***PTEN***	Phosphatase and tensin homolog	Involved in adiponectin-induced osteogenesis	miR-21-5p
***IL-10***	Interleukin 10	Inhibits bone resorption	miR-21-5p

## Supplementary Materials

The following are available online at http://www.mdpi.com/2311-553X/4/4/37/s1: Table S1: extended clinical characteristics of T1D patients, Table S2: R and P values of correlations between microRNAs expression and main bone parameters, Table S3: KEGG pathway analysis for validated target genes of miR-148a and miR-21-5p, Figure S1: correlation analysis between miR-148a and miR-21-5p expression and clinical characteristics of patients, Figure S2: correlation analysis between main parameters of bone metabolism and six selected miRNAs expression.
