# Supplementary material for: Serum Levels of miR-148a and miR-21-5p Are Increased in Type 1 Diabetic Patients and Correlated with Markers of Bone Strength and Metabolism
Source: Noncoding RNA. 2018 Nov 27;4(4):37. doi: 10.3390/ncrna4040037 (PMC6315714; doi:10.3390/ncrna4040037)

Table S1. Extended clinical characteristics of T1D patients. Presence of Diabetic complications, smoking habits, current medications, chronic inflammatory diseases alongside with microRNAs expression levels (reported as normalized $2^{-\Delta CT}$ values) for each patients analyzed have been reported in the Table below.

	Diabetic Complications	Smoking habits	Reported Drugs	Other Chronic Inflammatory Diseases	Micro Alb	Macro Alb	miR-148a-3p	miR-21-5p	miR-24-3p	miR-27a	miR-214	miR-375
T1D-1	none	none	none	none	none	none	0,0446	0,3090	0,1265	0,0028	0,0640	0,2784
T1D-2	none	none	none	none	none	none	0,0316	0,1270	0,1614	0,0350	0,0540	0,0073
T1D-3	none	none	none	none	none	none	0,0000	0,1280	0,1654	0,0016	0,0550	0,0001
T1D-4	retinopathy	none	none	none	none	none	0,0392	0,1080	0,1285	0,0125	0,0770	0,0015
T1D-5	none	none	E/P	none	none	none	0,0126	0,2050	0,1877	0,0508	0,0560	0,0032
T1D-6	none	none	depakin	none	none	none	0,0273	0,1510	0,0357	0,0169	0,0060	0,0043
T1D-7	none	yes	none	none	none	none	0,0000	0,3930	0,0836	0,0095	0,0210	0,0002
T1D-8	none	none	finasteride (alopecia)	none	none	none	0,0403	0,1910	0,1907	0,0507	0,0140	0,0035
T1D-9	none	none	none	none	none	none	0,0000	0,3470	0,0956	0,0507	0,0470	0,0033
T1D-10	none	none	none	none	none	none	0,0044	0,0850	0,1322	0,0031	0,0100	0,0786
T1D-11	retinopathy	none	salazopyrin	Ulcerative Colitis	none	none	0,0407	0,2500	0,1561	0,0276	0,0120	0,0742
T1D-12	none	yes	eutirox, rosuvastatin	Tyroiditis	none	none	0,0462	0,2440	0,0407	0,0046	0,0230	0,0002
T1D-13	none	none	none	Tyroiditis	none	none	0,0000	0,1400	0,3078	0,0099	0,1480	0,0032
T1D-14	none	yes	eutirox, cardioaspirin	Tyroiditis	none	none	0,0091	0,3820	0,7991	0,0206	0,0240	0,2891
T1D-15	none	none	none	none	none	none	0,01	0,31	0,25	0,05	0,04	0,00

Table S2. R and P values of correlations between microRNAs expression (miR-21-5p, miR-24-3p, miR-27a, miR-214, miR-375, miR-148a) evaluated as $2^{-\Delta Ct}$ and main bone metabolism parameters (BMD TB, BMC TB, BMC FEM, PTH, BMD L, BMC L, BMC N, BMD FEM, Osteocalcin) in T1D patients and non-diabetic subjects.

	**BMD TB**		**BMC TB**		**BMC FEM**		**PTH**		**BMD L**		**BMC L**		**BMC N**		**BMD N**		**BMD FEM**		**OSTEOCALCIN**	
	**r**	**p**	**r**	**p**	**r**	**p**	**r**	**p**	**r**	**p**	**r**	**p**	**r**	**p**	**r**	**p**	**r**	**p**	**r**	**p**
**miR-21-5p**	-0,17	0,37	-0,21	0,27	***-0,37***	***0,05***	0,34[illegible]	0,06	0,06	0,76	-0,01	0,96	-0,29	0,12	-0,27	0,15	-0,26	0,16	0,16	0,4
**miR-24-3p**	0,06	0,74	0,06	0,7	-0,13	0,5	0,09	0,6	0,31	0,09	0,25	0,18	0,08	0,65	-0,12	0,54	-0,05	0,77	-0,29	0,11
**miR-27a**	-0,12	0,54	-0,11	0,57	-0,11	0,57	0,16	0,39	-0,09	0,61	-0,08	0,68	-0,26	0,17	-0,23	0,23	-0,13	0,49	-0,11	0,56
**miR-214**	-0,27	0,16	-0,1	0,57	-0,21	0,28	0,35	0,06	-0,04	0,83	-0,13	0,48	-0,31	0,1	-0,285	0,133	-0,25	0,19	-0,15	0,43
**miR-375**	0,03	0,87	0,06	0,74	0,23	0,22	***0,45***	***0,013[illegible]***	-0,31	0,09	-0,28	0,14	0,22	0,25	-0,006	0,97	-0,009	0,96	0,17	0,37
**miR-148a**	***-0,37***	***0,04***	-0,25	0,19	-0,22	0,24	***0,39***	***0,033***	0,23	0,21	0,28	0,14	0,24	0,2	0,22	0,23	0,22	0,23	0,127	0,5

Table S3. KEGG pathways identified through bioinformatic analysis for both hsa-miR-148a-3p and hsa-miR-21-5p, are indicated with their relative p value, number of targeted genes and the targeting miRNA/s. Among these, we importantly found FoxO signaling pathway (targeted by both hsa-miR-148a-3p and hsa-miR-21-5p) and TGF-β signaling pathway (targeted by hsa-miR-148a-3p).

#	KEGG pathway	p-value	#genes	Targeting miRNA
1.	Prion diseases (hsa05020)	1,11E-10	3	hsa-miR-148a-3p
2.	Fatty acid biosynthesis (hsa00061)	4,44E-10	2	hsa-miR-148a-3p
3.	Proteoglycans in cancer (hsa05205)	3,86E-01	56	hsa-miR-148a-3p<br>hsa-miR-21-5p
4.	Lysine degradation (hsa00310)	8,99E-01	15	hsa-miR-148a-3p<br>hsa-miR-21-5p
5.	Fatty acid elongation (hsa00062)	6,45E+01	6	hsa-miR-21-5p
6.	Steroid biosynthesis (hsa00100)	7,72E+01	6	hsa-miR-148a-3p
7.	p53 signaling pathway (hsa04115)	0.00059	25	hsa-miR-148a-3p<br>hsa-miR-21-5p
**8.**	**FoxO signaling pathway (hsa04068)**	**0.00087**	**44**	**hsa-miR-148a-3p**<br>**hsa-miR-21-5p**
9.	Pathways in cancer (hsa05200)	0.00110	49	hsa-miR-21-5p
10.	Cell cycle (hsa04110)	0.00119	39	hsa-miR-148a-3p<br>hsa-miR-21-5p
11.	Chronic myeloid leukemia (hsa05220)	0.00134	19	hsa-miR-148a-3p
12.	Viral carcinogenesis (hsa05203)	0.00159	38	hsa-miR-148a-3p
13.	Colorectal cancer (hsa05210)	0.00220	13	hsa-miR-21-5p
14.	Thyroid hormone signaling pathway (hsa04919)	0.00223	19	hsa-miR-21-5p
15.	Hepatitis B (hsa05161)	0.00242	42	hsa-miR-148a-3p<br>hsa-miR-21-5p
16.	Oocyte meiosis (hsa04114)	0.00355	22	hsa-miR-148a-3p
17.	Fatty acid metabolism (hsa01212)	0.00429	7	hsa-miR-21-5p
18.	Transcriptional misregulation in cancer (hsa05202)	0.00470	39	hsa-miR-148a-3p<br>hsa-miR-21-5p
**19.**	**TGF-beta signaling pathway (hsa04350)**	**0.0051**	**15**	**hsa-miR-148a-3p**
20.	Hippo signaling pathway (hsa04390)	0.0140	23	hsa-miR-21-5p
21.	Fatty acid degradation (hsa00071)	0.0228	6	hsa-miR-21-5p
22.	Renal cell carcinoma (hsa05211)	0.0230	13	hsa-miR-148a-3p
23.	Sulfur metabolism (hsa00920)	0.0246	2	hsa-miR-148a-3p
24.	Glioma (hsa05214)	0.0260	14	hsa-miR-148a-3p
25.	Progesterone-mediated oocyte maturation (hsa04914)	0.0306	20	hsa-miR-148a-3p
26.	Endometrial cancer (hsa05213)	0.0365	11	hsa-miR-21-5p
27.	Biosynthesis of unsaturated fatty acids (hsa01040)	0.0394	4	hsa-miR-21-5p
28.	Thyroid cancer (hsa05216)	0.0414	8	hsa-miR-21-5p
29.	Prolactin signaling pathway (hsa04917)	0.0428	13	hsa-miR-21-5p

Figure S1. Correlation analysis between miR-148a and miR-21-5p expression, indicated as $2^{-\Delta Ct}$,and age (**a**) (**e**), BMI (**b**) (**f**) and disease duration (**c**) (**g**). Moreover, the expression of miR-148a and miR-21-5p has also been differentially analyzed between T1D patients with and without autoantibodies (AAb) (**d**) (**h**).

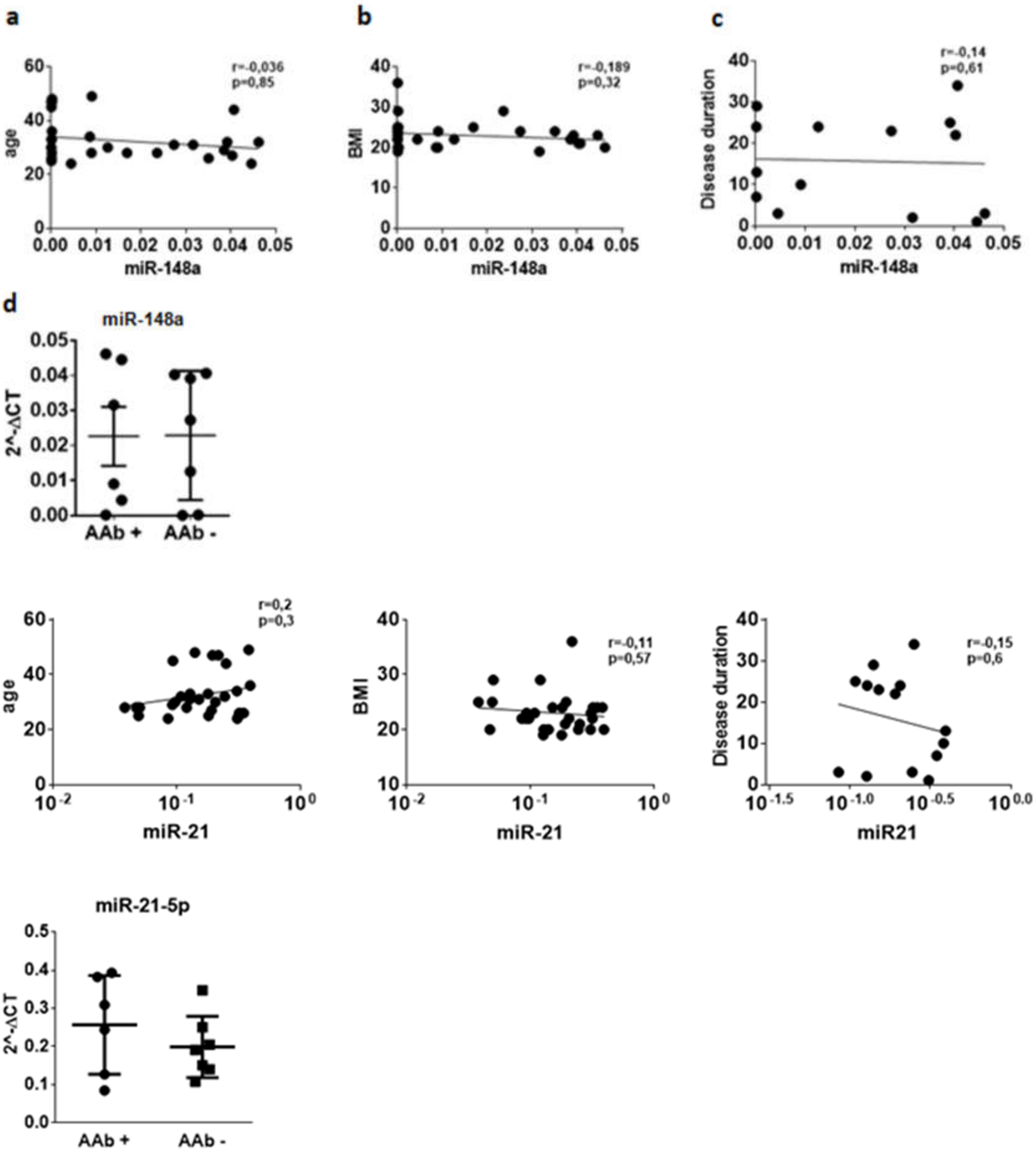

Figure S2. Correlation analysis between main parameters of bone metabolism (BMD TB, BMC TB, BMC FEM, PTH, BMD L, BMC L, BMC N, BMD N, BMD FEM and Osteocalcin) and the expression of miR-148a (**a**), miR-21-5p (**b**), miR-24 (**c**), miR-27a (**d**), miR-214 (**e**) and miR-375 (**f**), indicated as $2^{-\Delta Ct}$.

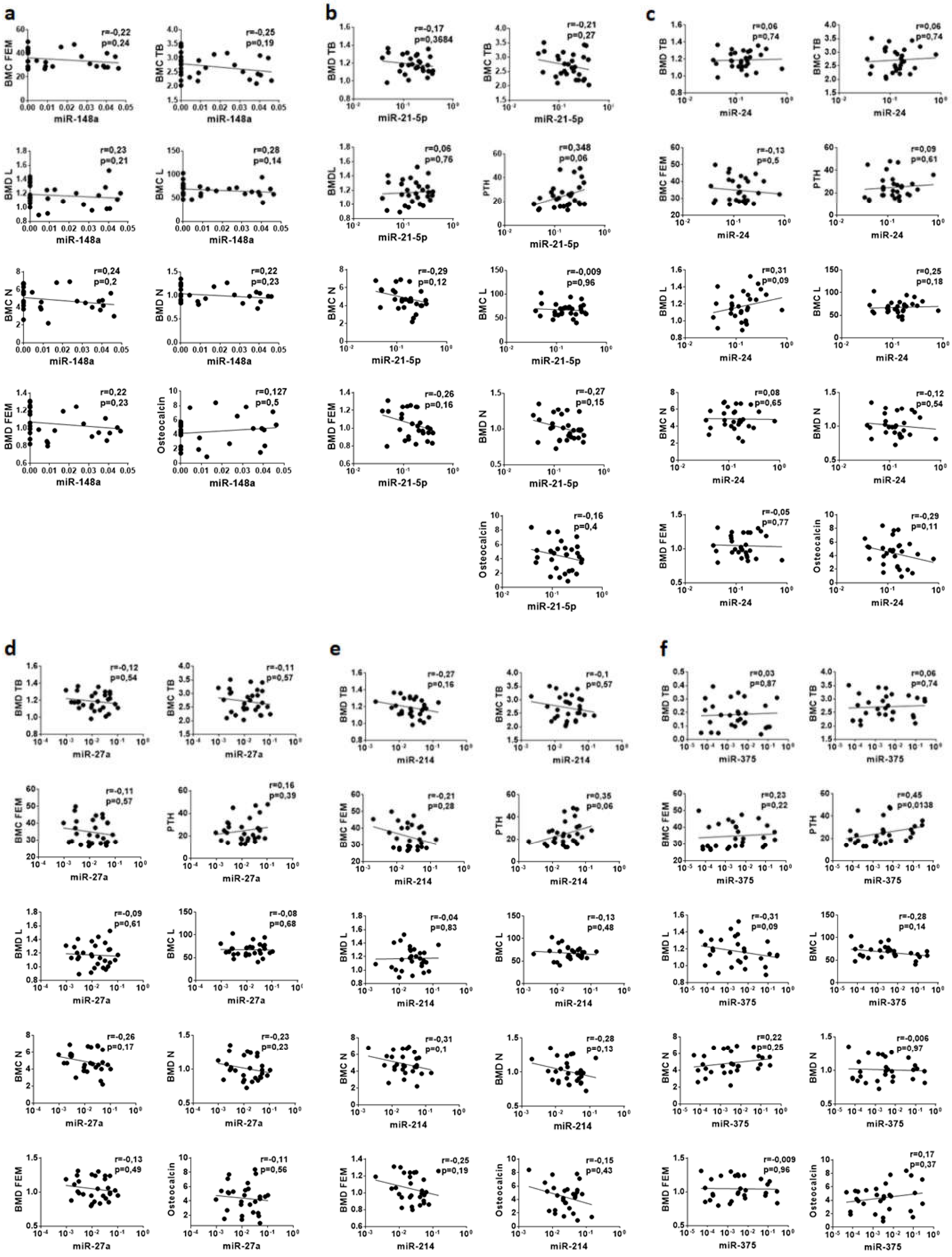

Supplement: Supplementary file 1 [file ncrna-04-00037-s001.pdf]
